# Time-to-event prediction analysis of patients with chronic heart failure comorbid with atrial fibrillation: a LightGBM model

**DOI:** 10.1186/s12872-021-02188-y

**Published:** 2021-08-04

**Authors:** Chu Zheng, Jing Tian, Ke Wang, Linai Han, Hong Yang, Jia Ren, Chenhao Li, Qing Zhang, Qinghua Han, Yanbo Zhang

**Affiliations:** 1 Department of Health Statistics, School of Public Health, Shanxi Medical University, 56 South Xinjian Road, Taiyuan, 030001 Shanxi Province China; 2 Department of Cardiology, The First Hospital of Shanxi Medical University, 85 South Jiefang Road, Taiyuan, 030001 Shanxi Province China; 3 Shanxi Provincial Key Laboratory of Major Diseases Risk Assessment, Taiyuan, 030001 Shanxi Province China

**Keywords:** Chronic heart failure, Atrial fibrillation, Mortality prediction, Risk stratification, LightGBM

## Abstract

**Background:**

Chronic heart failure (CHF) comorbid with atrial fibrillation (AF) is a serious threat to human health and has become a major clinical burden. This prospective cohort study was performed to design a risk stratification system based on the light gradient boosting machine (LightGBM) model to accurately predict the 1- to 3-year all-cause mortality of patients with CHF comorbid with AF.

**Methods:**

Electronic medical records of hospitalized patients with CHF comorbid with AF from January 2014 to April 2019 were collected. The data set was randomly divided into a training set and test set at a 3:1 ratio. In the training set, the synthetic minority over-sampling technique (SMOTE) algorithm and fivefold cross validation were used for LightGBM model training, and the model performance was performed on the test set and compared using the logistic regression method. The survival rate was presented on a Kaplan–Meier curve and compared by a log-rank test, and the hazard ratio was calculated by a Cox proportional hazard model.

**Results:**

Of the included 1796 patients, the 1-, 2-, and 3-year cumulative mortality rates were 7.74%, 10.63%, and 12.43%, respectively. Compared with the logistic regression model, the LightGBM model showed better predictive performance, the area under the receiver operating characteristic curve for 1-, 2-, and 3-year all-cause mortality was 0.718 (95%CI, 0.710–0.727), 0.744(95%CI, 0.737–0.751), and 0.757 (95%CI, 0.751–0.763), respectively. The net reclassification index was 0.062 (95%CI, 0.044–0.079), 0.154 (95%CI, 0.138–0.172), and 0.148 (95%CI, 0.133–0.164), respectively. The differences between the two models were statistically significant (*P* < 0.05). Patients in the high-risk group had a significantly higher hazard of death than those in the low-risk group (hazard ratios: 12.68, 13.13, 14.82, *P* < 0.05).

**Conclusion:**

Risk stratification based on the LightGBM model showed better discriminative ability than traditional model in predicting 1- to 3-year all-cause mortality of patients with CHF comorbid with AF. Individual patients’ prognosis could also be obtained, and the subgroup of patients with a higher risk of mortality could be identified. It can help clinicians identify and manage high- and low-risk patients and carry out more targeted intervention measures to realize precision medicine and the optimal allocation of health care resources.

## Perspectives

**What is known?**

The global prevalence of heart failure is estimated to exceed 37.7 million. The risk of mortality is higher in patients with CHF comorbid with AF, but few risk prediction tools are available for this subgroup.

**What is new?**

Using patients’ routine clinical variables, we designed and evaluated a risk stratification system based on the LightGBM model to effectively predict all-cause mortality in patients with CHF comorbid with AF. In this study, the LightGBM model performed better than the traditional risk prediction model.

## Introduction

Chronic heart failure (CHF) refers to a syndrome of ventricular filling or contraction disorders caused by damage to the structure and/or function of the heart under the influence of various pathogenic factors, leading to a series of complex clinical symptoms. In developed countries, patients with heart failure constitute about 1% to 2% of all adults, and this proportion increases to > 10% of adults aged > 70 years [1]. The global prevalence of heart failure is estimated to exceed 37.7 million. In the United States, the total medical cost of patients with heart failure was US$20.9 billion in 2012 and is expected to increase to US$53.1 billion by 2030 [2]. The high prevalence rate and poor prognosis of heart failure seriously affect patients’ physical and mental health and quality of life, and heart failure has become a global public health problem that threatens human health.

Atrial fibrillation (AF) is the most common arrhythmia in heart failure. AF increases the risk of thromboembolism (especially stroke) and may damage cardiac function, leading to deterioration of high-frequency symptoms. In the Framingham Heart Study, patients with heart failure comorbid with atrial fibrillation have a higher risk of mortality than those with only one disease [3]. The combination of AF and CHF is a major clinical burden because of the common pathophysiology, common risk factors, mutual causality, and poor prognosis of these concomitant diseases.

Accurate risk prediction can promote patient classification, assist clinicians in understanding individual patients’ disease risk, and preserve medical resources for patients with potential life-threatening needs in emergency care, thus delaying disease progression and improving the prognosis. However, the existing risk prediction models of heart failure have some shortcomings. First, traditional risk prediction models are based on the assumption that a linear relationship exists between variables and outcomes, which often limits their ability to model complex relationships. Second, the performance of the risk scores is still limited. For example, in the long-term heart failure registry of the European Society of Cardiology, the Meta-Analysis Global Group in Chronic Heart Failure risk score overestimated mortality while the Seattle Heart Failure Model underestimated mortality [4]; this limits their clinical application. Therefore, more accurate prognostic tools are needed.

The machine learning model can overcome the conditional limitations of the traditional survival prediction model, deal with high-dimensional interactions and nonlinear relationships between variables, improve the prediction ability of the model, and show better performance in identifying personalized outcome predictions [5]. It has been effectively used in heart disease research [6, 7], including the prediction of hospital readmission and mortality, etc. However, few prognostic studies have focused on the outcome of CHF comorbid with AF. Therefore, the goal of the present study was to identify the risk factors for all-cause mortality in patients with CHF comorbid with AF and to design and evaluate a LightGBM-based risk stratification model to predict 1- to 3-year all-cause mortality based on the patient’s baseline parameters at admission. It can help clinicians identify and manage high- and low-risk patients and carry out more targeted intervention measures to realize precision medicine and the optimal allocation of health care resources.

## Methods

### Data sources and study population

This is a prospective cohort study that involved patients who were hospitalized in the First Hospital of Shanxi Medical University and Shanxi Cardiovascular Hospital from January 2014 to April 2019 and diagnosed with CHF comorbid with AF. Patients were selected in strict accordance with the inclusion and exclusion criteria, and all patients provided written informed consent.

The inclusion criteria were an age of ≥ 18 years; typical symptoms (e.g., exertional or paroxysmal dyspnea, fatigue, or loss of appetite) or signs (e.g., edema of both lower extremities, rales in the lungs, or positive signs of hepatic jugular venous reflux) of CHF; New York Heart Association (NYHA) class of II to IV; current treatment with heart failure drugs or other treatment measures; and a history of AF or diagnosis of AF through clinical examination, standard electrocardiogram, and single-lead portable electrocardiogram monitoring.

The exclusion criteria were acute cardiovascular events in the past 2 months, concurrent mental illness, inability to understand or complete the questionnaire because of speech or intellectual impairment, and refusal to participate in the study.

### Data collection and predictor variables

According to the content of case records and heart failure guidelines [8], our group developed the chronic heart failure case report form (CHF-CRF) to collect the patients information. CHF-CRF included demographics (age, sex, family history, and other parameters), vital signs (blood pressure, body temperature, heart rate, and respiratory rate), causes of CHF [e.g. coronary heart disease (CHD), old myocardial infarction (OMI)], CHF comorbidities [chronic obstructive pulmonary disease (COPD), diabetes, atrial fibrillation, renal insufficiency and other conditions], symptoms and signs, laboratory test results included blood cell analysis, blood glucose, blood lipid, liver and kidney function, potassium, sodium, chlorine, B-type natriuretic peptide (BNP) and N-terminal pro B-type natriuretic peptide (NT-proBNP) et al., echocardiography was recorded along with standard and tissue Doppler imaging. LVEF was quantified by Simpson’s method. QRS duration was measured manually from limb leads using standard 12 lead ECG (25 mm/s). Drug therapy, percutaneous coronary intervention (PCI) and coronary artery bypass grafting (CABG) and other treatment information were also recorded on CHF-CRF.

### Outcomes

The patients were followed up at 1, 3, 6 and 12 months after discharge and annually thereafter. Patients with less than 3-year follow-up time were excluded, and the outcome was all-cause mortality within 1, 2, and 3 years, including death from heart failure, cardiovascular causes, and other causes. The death information was composed of two parts: one was that the follow-up personnel conduct regular follow-up of the patient, and the other was to inquire in the information system of the death cause registration report of Shanxi Province based on the patient's ID number.

### Data pre-processing

To make full use of clinical information, we filled in the missing data before variable screening, missing continuous variables were imputed with median, and missing categorical variables were imputed with mode. At the same time, BNP, coronary CT and coronary angiography results were excluded in order to exclude the influence of variables with high missing ratio on the prediction performance of the model. Estimated glomerular filtration rate was calculated by CKD-EPI using cystatin C [9].

### Machine Learning Modeling Approach: LightGBM

To solve the time-consuming shortcomings of the traditional boosting algorithm under big data, Ke et al. [10] proposed two novel techniques: Gradient-based One-Side Sampling (GOSS) and Exclusive Feature Bundling (EFB). GOSS retains all data with large gradients and randomly samples data with small gradients, thereby reducing the amount of calculation and optimizing speed and memory. EFB can bundle mutually exclusive features into a single feature to reduce the dimension of features. LightGBM is a new gradient boosting decision tree algorithm with GOSS and EFB.

### Model development and performance evaluation

The data set was randomly divided into a training set and test set at a 3:1 ratio. This process was repeated 100 times to ensure the stability of the model. In the training set, the SMOTE algorithm was used for data equalization sampling, and fivefold cross validation was used for LightGBM model training; a prediction performance evaluation was performed on the test set and compared using the logistic regression method.

The area under the receiver operating characteristic curve (AUC), accuracy, sensitivity, specificity, and *f*-measure were calculated to quantify the model’s discriminative ability in each year. Calibration of the model was evaluated by the Brier score, which is defined as the mean square difference between the observed outcomes and the predictions. The Hosmer–Lemeshow goodness-of-fit test of the model was visualized by calibration curve plots. The net reclassification index was used to quantify the degree of improvement in the prediction ability of the LightGBM algorithm compared with the logistic regression model.

### Risk groups

One of the data splits was randomly selected for training and testing of the model, and the receiver operating characteristic curves were plotted. Using the maximal Youden’s index as the best cut-off value, the 1-, 2-, and 3-year probabilities of death predicted by the LightGBM model were divided into high-risk and low-risk groups.

### Statistical analysis

Continuous variables are presented as median (interquartile range), and categorical variables are presented as number (percentage). To determine the factors related to all-cause mortality, the recursive feature elimination method was used for feature selection. The selected continuous variables and categorical variables were analyzed with the Mann–Whitney U test and chi-square test, respectively.

DeLong test was used to compare the AUC between models, and *P* < 0.05 was considered statistically significant. The survival rate was presented on a Kaplan–Meier curve and compared by a log-rank test, and the hazard ratio was calculated by a Cox proportional hazard model.

### Sensitivity analysis

Sensitivity analyses were performed using different subgroups, including heart failure type [heart failure with a reduced left ventricular ejection fraction (LVEF), midrange LVEF, or preserved LVEF], sex, and age (≤ 74 or ≥ 75 years). All statistical analyses were performed using Python 3.7.

## Results

### Baseline characteristics of patients

The baseline characteristics of patients with CHF comorbid with AF are shown in Table 1. In total, 1796 patients were included in this study. The median age of all patients in the entire cohort was 73 (64–80) years, and 63.42% were male. The most common comorbidity was hypertension (62.97%), followed by diabetes (28.56%). The 1-, 2-, and 3-year cumulative mortality rates of patients with CHF comorbid with AF were 7.74%, 10.63%, and 12.43%, respectively.

**Table 1:** Patients’ baseline characteristics (*n* = 1796)

Variables	Description
Age, *year*s	73 (64, 80)
Male, *n (*%)	1139 (63.42)
BMI, *kg/m*^*2*^	24.22 (22.04, 26.87)
SBP, *mmHg*	130 (116, 142)
DBP, *mmHg*	80 (70, 86)
Heart rate, *b.p.m*	75(64, 90)
LVEF, *%*	50 (40, 59)
LVD, *mm*	43 (39, 46)
*NYHA class, n (%)*	
II	574 (31.96)
III or IV	1222 (68.04)
*AF type*	
Paroxysmal	274(15.26)
Persistent	39(2.17)
Permanent	1483(82.57)
*Causes of HF*	
CHD	1609(89.59)
OMI	854(47.55)
*Comorbidities, n (%)*	
Hypertension	1131 (62.97)
Type II diabetes	513(28.56)
COPD	432 (24.05)
Stroke	486(27.06)
*Therapy, n (%)*	
Beta-blockers	1213(67.54)
ACEI/ARB	854 (47.55)
MRA	1321(73.55)
Loop diuretic	1252 (69.71)
Digitalis	486 (27.06)
Calcium antagonist	313 (17.43)
Anticoagulant	1646 (91.65)
PCI	365 (20.32)
CABG	110 (6.12)
Pacemaker	62 (3.45)
Defibrillator	7 (0.39)
CRT	6 (0.33)

BMI: body mass index, SBP: systolic blood pressure, DBP: diastolic blood pressure, LVEF: Left ventricular ejection fraction, LVD: left atrial dimension, NYHA: New York Heart Association, AF: atrial fibrillation, CHD: coronary heart disease, OMI: old myocardial infarction, COPD: chronic obstructive pulmonary disease, ACEI: angiotensin-converting enzyme inhibitor, ARB: angiotensin receptor blocker, MRA: mineralocorticoid receptor antagonist, PCI: percutaneous coronary intervention, CABG: coronary artery bypass grafting. CRT: Cardiac resynchronization therapy

### Predictor variable

The recursive feature elimination method based on the random forest model was used for feature screening. As shown in Table 2, the main predictors of all-cause mortality were older age; a higher white blood cell count (WBC), red blood cell distribution width (RDW), aspartate aminotransferase (AST) level, total bilirubin (TBIL) level, alkaline phosphatase (ALP), blood urea nitrogen (BUN) level, uric acid level, N-terminal pro-brain natriuretic peptide (NT-proBNP) level, and NYHA class; a lower body mass index (BMI), diastolic blood pressure (DBP), hemoglobin level, albumin level, estimated glomerular filtration rate was calculated using cysteine C level (CyscGFR) and left ventricular ejection fraction (LVEF); a wider QRS complex; the combination of COPD and diabetes; and not taking beta-blockers, and angiotensin-converting enzyme inhibitor (ACEI)/angiotensin receptor blocker (ARB).

**Table 2:** Predictor variables of all-cause mortality in the model

Variables	Survive (*n* = 1573)	Death (*n* = 223)	*P*
Age, *year*s	72 (63, 79)	77 (71, 82)	< 0.001
BMI, *kg/m*^*2*^	24.34(22.23,27.00)	23.52(20.83,25.95)	< 0.001
DBP, *mmHg*	80 (70,86)	76 (70, 84)	0.01
WBC,*10*^*9*^*/L*	6.60 (5.30, 7.80)	7.00 (5.80, 8.60)	< 0.001
Hemoglobin, *g/L*	135 (125, 150)	129 (118, 142)	< 0.001
RDW, %	14.1 (13.54, 14.56)	14.5 (13.93, 15.26)	< 0.001
AST, *U/L*	23.00 (21.00, 24.00)	25.00 (21.00, 39.00)	< 0.001
Albumin, *U/L*	42.00 (39.80, 45.40)	40.00 (37.00, 43.00)	< 0.001
TBIL, *umol/L*	16.50 (12.70, 20.20)	20.20 (14.60, 22.80)	< 0.001
ALP, *U/L*	76 (64, 88)	76 (64, 97)	0.029
BUN, *mmol/L*	6.50 (5.19, 8.20)	7.81 (5.80,10.20)	< 0.001
CyscGFR, *ml/min/1.73m*^*2*^	57.70(51.25,69.77)	46.40(38.69,61.03)	< 0.001
Uric acid, *umol/L*	399.0 (327.0, 470.0)	457.0 (350.0, 523.0)	< 0.001
NT-proBNP, *ng/L*	2201(983, 3293)	4563 (2201,7532)	< 0.001
QRS, *ms*	98 (88, 116)	106 (90, 130)	< 0.001
LVEF, %	50 (40, 59)	45 (38, 56)	< 0.001
*NYHA class, n (%)*			
II	560 (97.56)	14 (2.44)	< 0.001
III or IV	1013(82.90)	209 (17.10)	
*COPD n (%)*			
No	1242 (91.06)	122(8.94)	< 0.001
Yes	331 (76.62)	101 (23.38)	
*Diabetes n (%)*			
No	1148 (89.48)	135 (10.52)	< 0.001
Yes	425 (82.85)	88 (17.15)	
*ACEI/ARB, n (%)*			
Yes	763 (89.34)	91(10.66)	0.031
No	810 (85.99)	132 (14.01)	
*Beta-blockers, n (%)*			
Yes	1112 (91.67)	101 (8.33)	< 0.001
No	461 (79.07)	122 (20.93)	

Data are presented as median (interquartile range) or *n* (%)

BMI: body mass index, DBP: diastolic blood pressure, WBC: white blood cell, RDW: red blood cell distribution width, ALT: alanine aminotransferase, AST: aspartate aminotransferase, TBIL: total bilirubin, BUN: blood urea nitrogen, ALP: alkaline phosphatase, CyscGFR: estimated glomerular filtration rate was calculated by cystatin C, NT-proBNP: N-terminal pro-brain natriuretic peptide, LVEF: left ventricular ejection fraction, NYHA: New York Hearth Association, COPD: chronic obstructive pulmonary disease, ACEI: angiotensin-converting enzyme inhibitor, ARB: angiotensin receptor blocker

### Model prediction performance

**Fig. 1:**
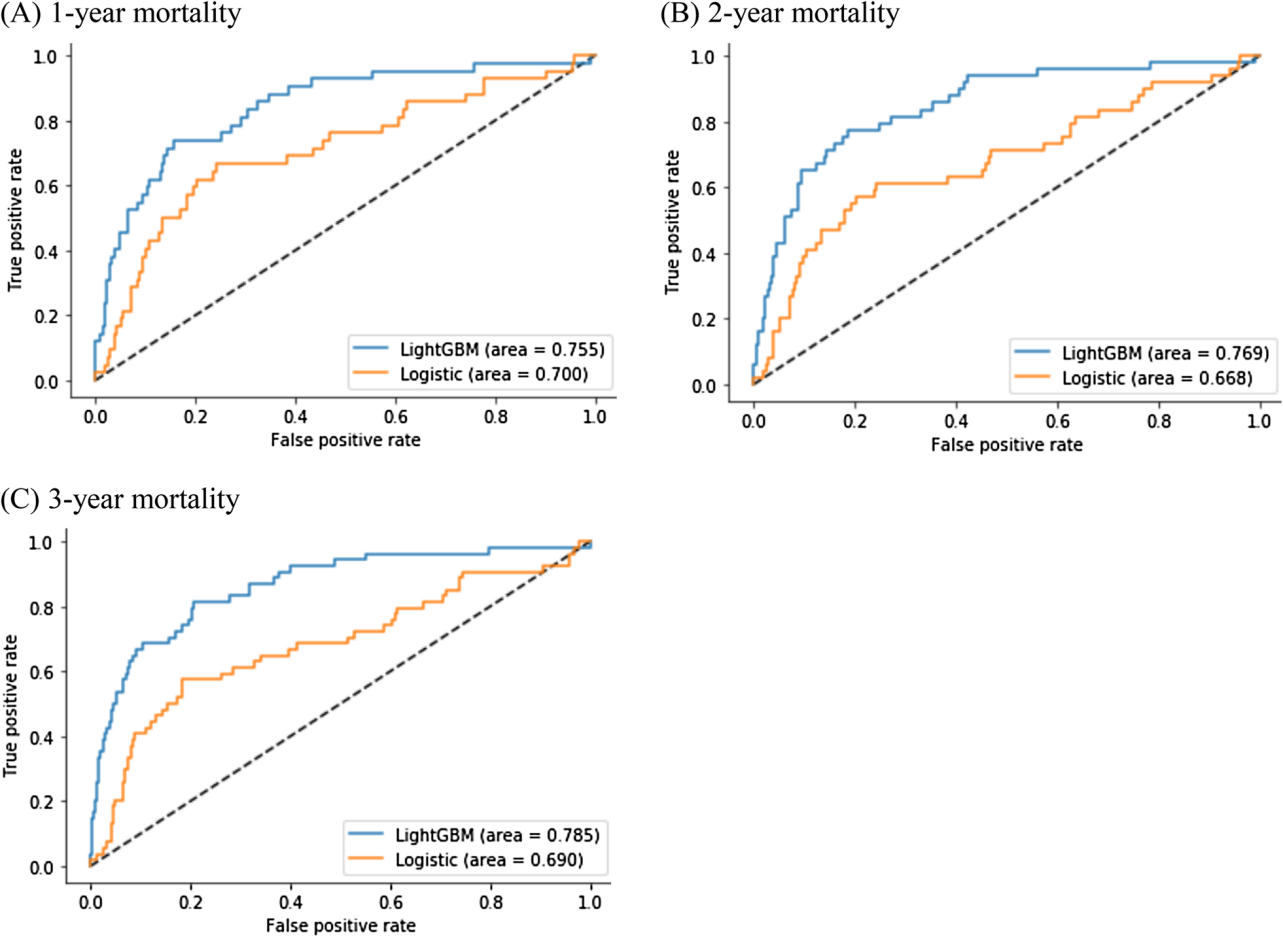
Receiver operating characteristic curves of different models

Compared with logistic regression, the LightGBM model exhibited higher discrimination and lower Brier score in the 1-, 2-, and 3-year follow-up of the test cohort (Table 3, Fig. 1). The prediction accuracy of all-cause mortality of the LightGBM model was 0.853 (95%CI, 0.850–0.857), 0.855 (95%CI, 0.852–0.859), and 0.864(95%CI, 0.861–0.868), and the AUC was 0.718 (95%CI, 0.710–0.727), 0.744 (95%CI, 0.737–0.751), and 0.757 (95%CI, 0.751–0.763), respectively, and Brier score was 0.146 (95%CI, 0.143–0.150), 0.145 (95%CI, 0.141–0.148), and 0.135 (95%CI, 0.132–0.138), respectively. The prediction accuracy of all-cause mortality of the Logistic regression model was 0.709(95%CI, 0.705–0.713), 0.715(95%CI, 0.710–0.720), and 0.732 (95%CI, 0.727–0.737), and the AUC was 0.687 (95%CI, 0.680–0.694), 0.667 (95%CI, 0.660–0.673) and 0.683 (95%CI, 0.677- 0.689), respectively, and Brier score was 0.291 (95%CI, 0.287–0.295), 0.285 (95%CI, 0.280–0.290), and 0.268 (95%CI, 0.263–0.273), respectively.

**Table 3:** Comparison of prediction performance of different classification models

	Logistic regression			LightGBM		
	1-year	2-year	3-year	1-year	2-year	3-year
AUC	0.687 (0.680,0.694)	0.667 (0.660, 0.673)	0.683 (0.677, 0.689)	0.718* (0.710, 0.727)	0.744* (0.737, 0.751)	0.757* (0.751, 0.763)
Accuracy	0.709 (0.705, 0.713)	0.715 (0.710, 0.720)	0.732 (0.727, 0.737)	0.853 (0.850, 0.857)	0.855 (0.852, 0.859)	0.864 (0.861, 0.868)
Sensitivity	0.662 (0.646, 0.677)	0.606 (0.593, 0.619)	0.618 (0.605, 0.631)	0.559 (0.542, 0.576)	0.603 (0.589, 0.617)	0.615 (0.603, 0.626)
Specificity	0.713 (0.708, 0.717)	0.727 (0.722, 0.734)	0.748 (0.742, 0.754)	0.878 (0..875, 0.881)	0.885 (0.882, 0.888)	0.900 (0.897, 0.903)
*f*-measure	0.257 (0.250, 0.264)	0.308 (0.300, 0.315)	0.363 (0.355, 0.371)	0.367 (0.356, 0.378)	0.465 (0.455,0.475)	0.528 (0.519, 0.537)
Brier score	0.291 (0.287, 0.295)	0.285 (0.280, 0.290)	0.268 (0.263,0.273)	0.146 (0.143, 0.150)	0.145 (0.141, 0.148)	0.135 (0.132, 0.138)

Data are presented as mean (95% confidence interval), CI: confidence interval, AUC: area under the curve

^*^DeLong test, *P* < 0.05, the AUC of 1-, 2-, and 3-year all-cause mortality were different between LightGBM and logistic regression model

**Fig. 2:**
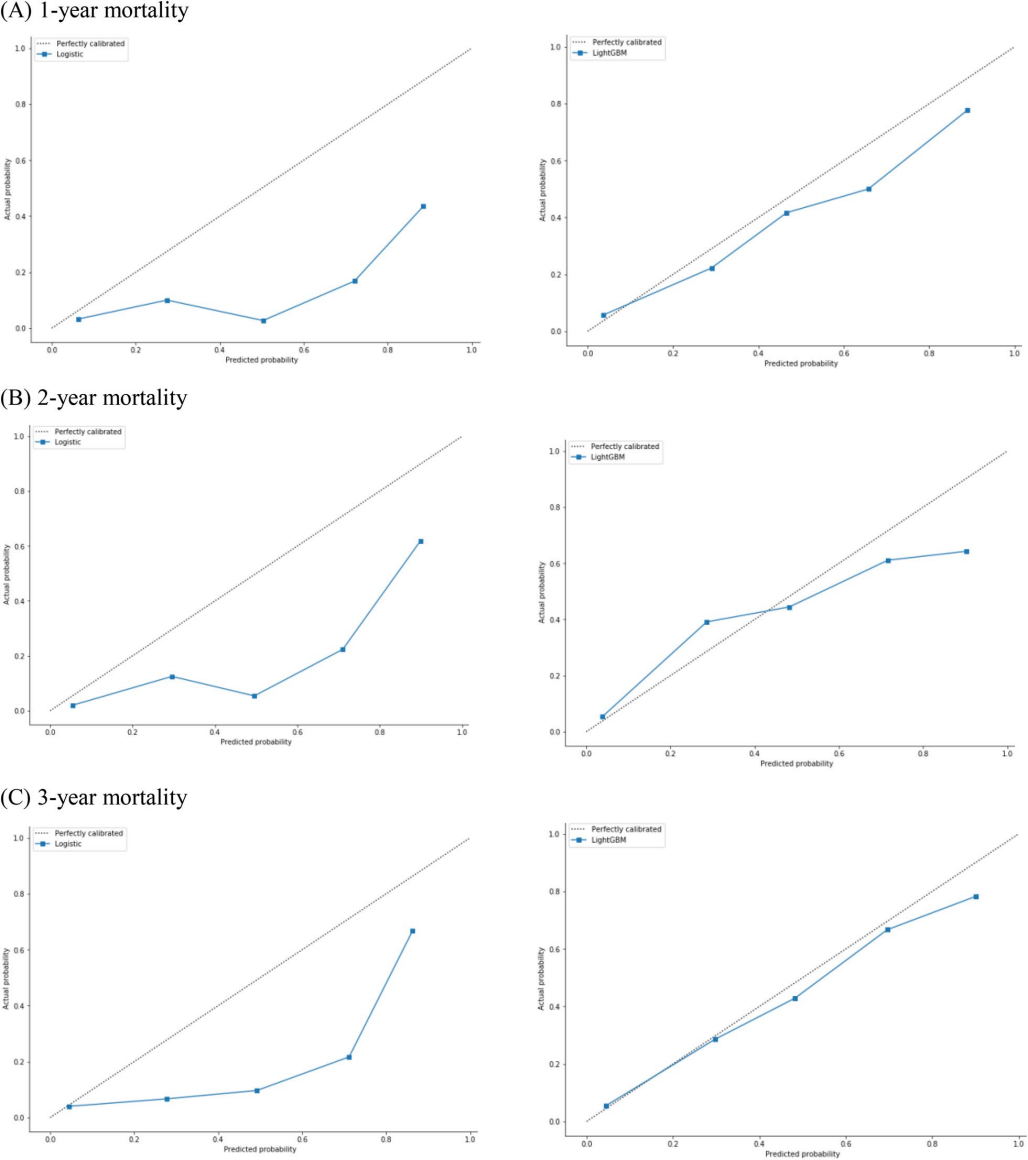
Calibration plots for 1-, 2- and 3-year all-cause mortality outcome (on the left is the logistic regression model, and on the right is the LightGBM model. The horizontal axis of the calibration plot is the predicted probability, the vertical axis is the true probability, and the 45-degree straight line represents the perfect prediction line.)

The calibration curve plots indicated that the LightGBM model was generally well calibrated, with intercepts closer to 0 and slopes closer to 1, while logistic regression showed poor calibration (Fig. 2).

The classification improvement of each year was calculated and compared with the logistic regression model. The net reclassification index of the LightGBM model in the 1-, 2-, and 3-year follow-up was 0.062 (95%CI, 0.044–0.079, *P* < 0.05), 0.154 (95%CI, 0.138–0.172, *P* < 0.05), and 0.148 (95%CI, 0.133–0.164, *P* < 0.05), respectively, suggesting that the mortality prediction ability of the LightGBM model was better than that of logistic regression.

### Feature importance

**Fig. 3:**
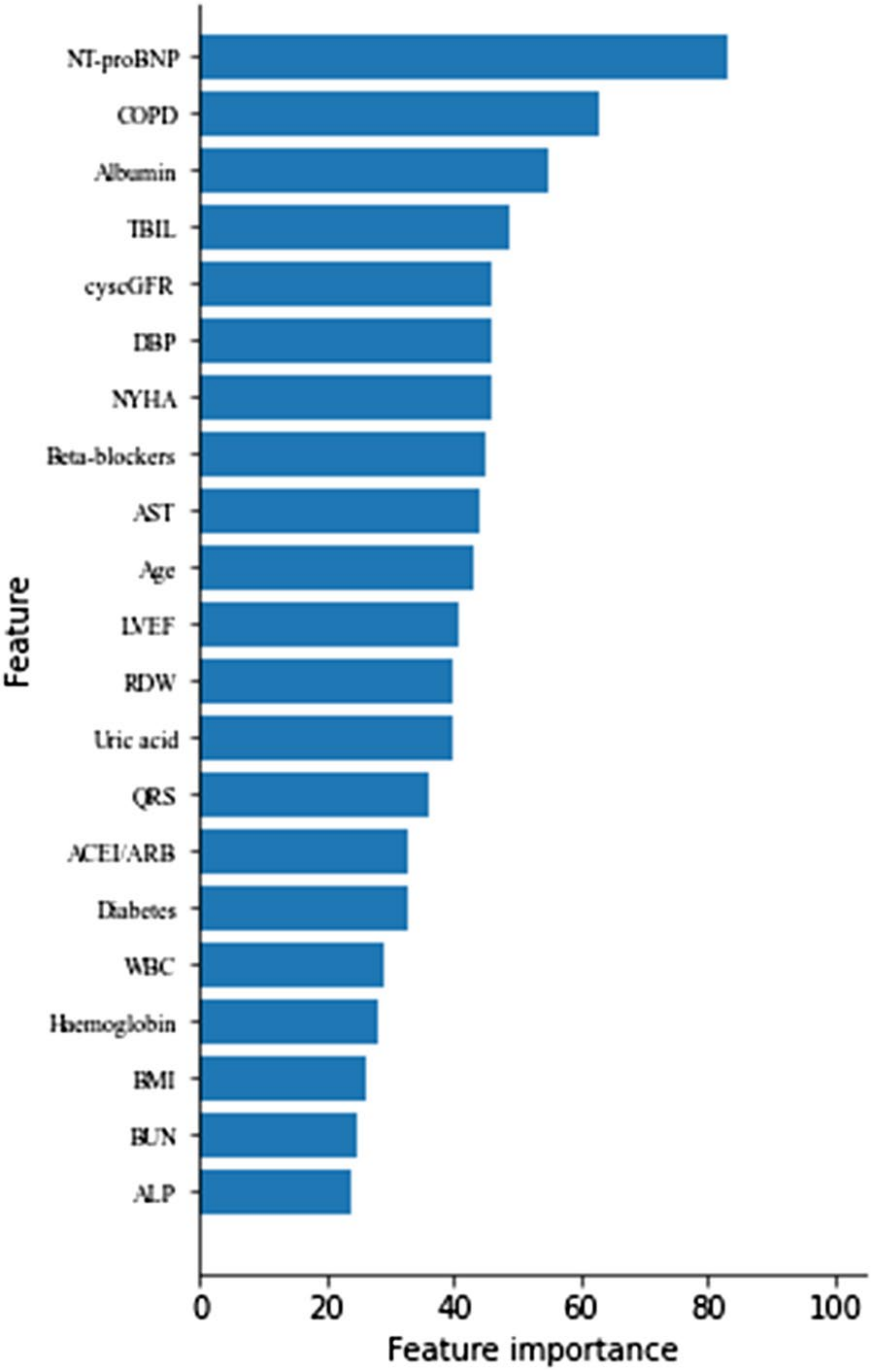
Feature importance of all-cause mortality

The feature importance of all-cause mortality is shown in Fig. 3. The importance of each feature was quantified by the number of times a feature was used to split in the model, and a higher value of feature importance was associated with a greater contribution to the risk prediction of the model. The importance of the first 11 features was ranked as follows: NT-proBNP level, COPD, albumin level, TBIL level, CyscGFR level, DBP, NYHA class, beta-blockers, AST level, age, and LVEF.

### LightGBM model-based risk stratification

The probabilities of death predicted by the LightGBM model were divided into high-risk and low-risk groups, using the maximal Youden’s index as the best optimal cut-off value (0.492, 0.498, 0.497, respectively). At each cut-off, the sensitivity and specificity of model prediction were 0.738 and 0.843, 0.776 and 0.815, 0.815 and 0.795, respectively.

**Fig. 4:**
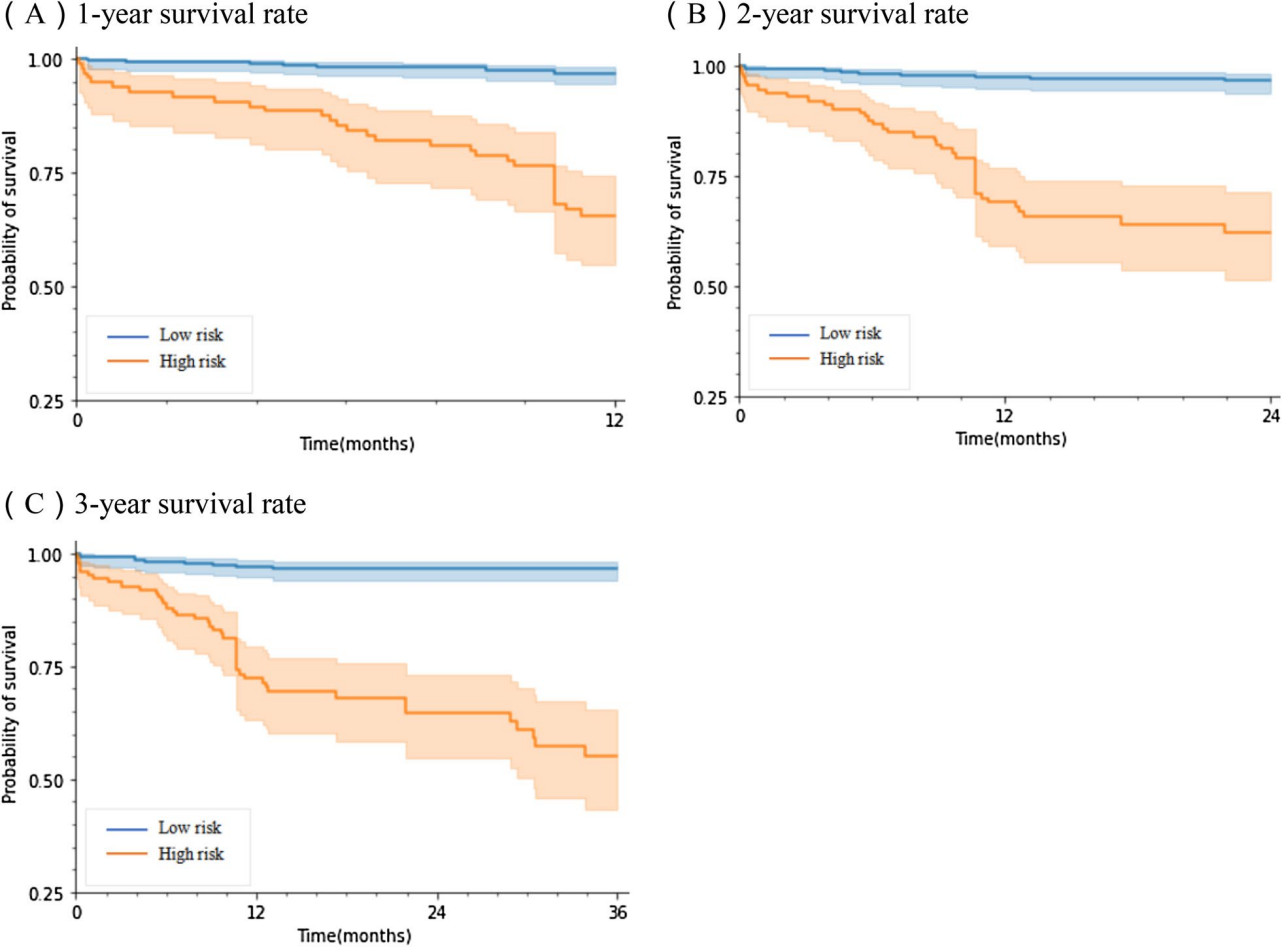
Kaplan–Meier curves and log-rank test (*P* < 0.001)

As shown by the Kaplan–Meier curve, log-rank test, and Cox proportional hazards model, there were significant differences in the distribution of death events between the two groups in all follow-up years (Fig. 4, Table 4). Patients in the high-risk group had a significantly higher hazard of death than those in the low-risk group, the hazard ratio was 12.68, 13.13, 14.82, respectively.

**Table 4:** Hazard ratios of all-cause mortality in Cox proportional hazards model

Time	*b*_*j*_	*s*_*b*_	Wald $\chi^{2}$	*P*	*HR* (95%CI)
1-year	2.54	0.35	7.22	< 0.005	12.68 (6.36, 25.25)
2-year	2.57	0.34	7.46	< 0.005	13.13 (6.69, 25.76)
3-year	2.70	0.35	7.66	< 0.005	14.82 (7.43, 29.56)

### Results of the sensitivity analysis

The models in each subgroup performed well, and the predictive performance between the sexes was similar. For 1-year mortality, however, the discrimination was lower for patients aged ≥ 75 years. For example, the model had a discrimination of 0.693 for patients aged ≥ 75 years and 0.761 for patients aged ≤ 74 years (Table 5).

**Table 5:** Subgroup-Specific ROC of the LightGBM Models

Subgroup	1 year	2 years	3 years
HFrEF	0.721 (0.703, 0.739)	0.743 (0.731, 0.756)	0.758 (0.746, 0.769)
HFmrEF	0.760 (0.746, 0.774)	0.765 (0.754, 0.775)	0.764 (0.754, 0.773)
HFpEF	0.711 (0.700, 0.722)	0.722 (0.712, 0.732)	0.730 (0.721, 0.739)
Female	0.756 (0.744, 0.769)	0.741 (0.729, 0.752)	0.753 (0743, 0.763)
Male	0.749 (0.741, 0.758)	0.751 (0.743, 0.759)	0.763 (0.755, 0.770)
Age ≤ 74 years	0.761 (0.751, 0.772)	0.757 (0.747, 0.767)	0.762 (0.753,0.770)
Age ≥ 75 years	0.693 (0.684, 0.701)	0.708 (0.700, 0.716)	0.728 (0.720, 0.736)

HFrEF: heart failure with reduced ejection fraction, HFmrEF: heart failure with midrange ejection fraction, HFpEF: heart failure with preserved ejection fraction

## Discussion

In this study, we designed and evaluated a risk stratification system based on the LightGBM model to predict 1- to 3-year all-cause mortality in patients with CHF comorbid with AF. The risk stratification system showed moderate predictive performance with an average AUC of 0.740. CHF and AF are causes and effects of each other. Damage to the cardiac structure or function, abnormal activation of neurohumoral mechanisms, and remodeling of ion channels in patients with CHF can lead to myocardial remodeling, enlarge the atrium, change the electrical activity characteristics of atrial myocytes, and promote the occurrence and persistence of AF. Similarly, regular atrial contraction loss and irregular electrocardiographic conduction in patients with AF can lead to the impairment of left ventricular diastolic and systolic function and promote the occurrence and development of heart failure. Previous studies have shown that the existence of AF is related to the poor prognosis of CHF [11]. However, identifying the risk factors of adverse prognosis of CHF comorbid with AF and taking effective control and treatment measures will help to reduce the incidence of adverse events such as death.

Consideration of the relationship between multiple clinical variables of a single patient and mortality is a great challenge for clinicians, and it is often easy to ignore the potential relationship between variables. ML methods can handle complex interactions and nonlinear relationships between predictors, allowing the selection of unknown variables and the best predictive subset of the model through continuous iteration. As an emerging algorithm in machine learning, the LightGBM algorithm overcomes the limitations of traditional boosting algorithms. LightGBM algorithm has the following advantages: first, it has faster training speed, higher efficiency and better accuracy; Second, it has lower memory consumption and can process large-scale data; Third, it supports parallel, distributed, and GPU learning. Experiments show that LightGBM algorithm can speed up the training process of traditional gradient boosting decision tree (GBDT) by more than 20 times, while achieving almost the same accuracy, and LightGBM can be significantly better than the extreme gradient boosting (XGBoost) algorithm and the stochastic gradient boosting (SGB) algorithm in computing speed and memory consumption [10]. Therefore, we chose LightGBM for this research.

We screened the most important predictors of all-cause mortality in the cohort of this study. According to the feature importance ranking, older age; a higher NT-proBNP level, NYHA class, AST level, TBIL level; a lower DBP level, albumin level, cyscGFR and LVEF; combined with COPD; and not taking beta-blockers had a relatively large contribution to prediction of the risk of death in patients with CHF comorbid with AF.

Our study found that NT-proBNP is an important predictor of prognosis in patients with CHF comorbid with AF. The NT-proBNP level is positively correlated with the severity of heart failure, and is closely related to NYHA class, end-diastolic pressure, and degree of hemodynamic disturbances, and can be used as an effective means of prognostic evaluation [12, 13]. Abnormal liver enzymes often appear in patients with heart failure, with a prevalence of 30–60% [14]. Increased venous congestion and impaired hemodynamics are common causes of abnormal liver enzymes in patients with heart failure. Abnormal liver function may lead to increased fluid overload due to hypoalbuminemia and low-osmolality state, which may lead to deterioration of heart failure. This explains the liver function indicators as powerful predictors of prognosis in patients with CHF comorbid with AF in our study, and is consistent with other studies [15–17].

Renal dysfunction is a common complication of CHF, the pathophysiology of cardiorenal syndrome is closely related to decreased cardiac output and increased central venous pressure. About 40% of hospitalized patients with heart failure showed elevated serum creatinine and decreased glomerular filtration rate (GFR) [18]. Cystatin C is considered to be a more sensitive blood marker of renal function than creatinine and is less strongly affected by muscle mass, age, sex, or race. The CyscGFR is closer to the directly measured glomerular filtration rate and has better prognostic value [19]. Renal function parameters were found to be predictors of adverse events in patients with CHF comorbid with AF in the present study, consistent with previous reports [20–23].

Diabetes (28.12%) and COPD (24.05%) are common complications and predictors of poor prognosis in the present study. COPD and AF have common risk factors and therefore often coexist. COPD greatly limits the survival of patients. Previous study has found that patients with concurrent AF and COPD have higher cardiovascular mortality and all-cause mortality [24]. The prevalence of diabetes is 12–44% in heart failure patients, depending on the severity of heart failure and whether the left ventricular ejection fraction is reduced. Diabetes is a powerful independent predictor of death in patients with advanced heart failure [25]. Type II diabetes can cause inflammation of adipose tissue, and the resulting systemic inflammation can lead to the expansion of epicardial adipose tissue and proinflammatory transformation. In one study, patients with multiple non-cardiovascular comorbidities had a higher risk of competitive death [26]. The white blood cell count is elevated in patients with AF and CHF, and the increase of inflammatory markers in patients with cardiovascular disease (especially heart failure) can be considered a factor for a poor prognosis [27]. There is a lot of evidence that systemic inflammation is present in COPD patients [28]. Combined with the above, it may explain to some extent the cause of high mortality in patients with COPD or diabetes. The RDW is the coefficient of variation of the red blood cell volume and reflects the heterogeneity of the red blood cell volume. It is a proven predictor of adverse outcomes of heart failure [29].

In the present study, patients with a higher BMI had a lower prognostic risk. This seems to reflect the obesity paradox but laterally indicates that the BMI is not an independent predictor of CHF comorbid with AF. Lower DBP is associated with an increased risk of adverse cardiovascular events in patients with heart failure with a preserved LVEF [30, 31], and our study revealed a similar relationship in patients with CHF comorbid with AF. Oral beta-blocker therapy is helpful to control the heart rate. In Chinese elderly patients with heart failure, admission without beta-blocker therapy is a specific independent risk factor for readmission or death within 1 year [32]. Previous studies have also shown a moderate association between the use of ACEI/ARB and lower mortality [33].

Based on the above predictive variables, we conducted subgroup analysis, but found that LightGBM model had a low discrimination for patients aged ≥ 75 years. We speculate that the possible reason is that patients aged ≥ 75 years have more complicated conditions, more complications, and are more likely to have some complex clinical emergencies. The prediction model constructed by conventional inspection indexes can not achieve good prediction results, and we have found the same results in another machine learning study[34].

Compared with previous reports, the innovations of this study are the use of patients with CHF comorbid with AF as the target population; the use of the LightGBM as a new machine learning prediction model; inclusion of a variety of non-cardiac clinical variables such as COPD, diabetes, and liver and kidney function in the model; and the fact that the patients’ clinical variables were easy to obtain. Compared with the traditional risk prediction model, the LightGBM model performs better in predicting all-cause mortality in patients with CHF comorbid with AF. It can risk-stratify individuals and identify patients with a high risk of death during the whole follow-up period. Patients in the high-risk group had a significantly higher hazard of death than those in the low-risk group, the hazard ratio was 12.68, 13.13, 14.82 in our study, respectively. Clinicians can carry out active intervention programs for high-risk patients and controllable variables to improve patients’ quality of life and reduce mortality. In addition, we performed 100 random splits on the data set to ensure that the prediction results of the model were more robust.

## Limitations

Our study has two main limitations. First, we did not perform external verification; the external accuracy of the model may need to be confirmed by further research. Second, we did not include genetic biomarkers (such as microRNA); thus, the clinical data and biomarkers of patients with CHF should be combined in future studies to establish a new predictive model with comprehensive patient information to improve prognostic risk assessment.

## Conclusions

Using patients’ routine clinical variables, we designed and evaluated a risk stratification system based on the LightGBM model to effectively predict all-cause mortality in patients with CHF comorbid with AF and identify subgroups of patients with a high risk of death. It can help clinicians identify and manage high- and low-risk patients and carry out more targeted intervention measures to realize precision medicine and the optimal allocation of health care resources.

## Data Availability

The datasets used and/or analyzed during the current study are available from the corresponding author on reasonable request.

## Abbreviations

CHF: Chronic heart failure
COPD: Chronic obstructive pulmonary disease
GOSS: Gradient-based One-Side Sampling
EFB: Exclusive Feature Bundling
ROC: Receiver operating characteristic curve
AUC: Area under the receiver operating characteristic curve
LVEF: Left ventricular ejection fraction
LVD: Left atrial dimension
BMI: Body mass index
SBP: Systolic blood pressure
DBP: Diastolic blood pressure
NYHA: New York Heart Association
ACEI: Angiotensin-converting enzyme inhibitor
ARB: Angiotensin receptor blocker
PCI: Percutaneous coronary intervention
CABG: Coronary artery bypass grafting
MRA: Mineralocorticoid receptor antagonist
CRT: Cardiac resynchronization therapy
WBC: White blood cell
RDW: Red blood cell distribution width
ALT: Alanine aminotransferase
AST: Aspartate aminotransferase
TBIL: Total bilirubin
BUN: Blood urea nitrogen
ALP: Alkaline phosphatase
CyscGFR: Estimated glomerular filtration rate was calculated by cystatin C
NT-proBNP: N-terminal pro-brain natriuretic peptide
CI: Confidence interval
HFrEF: Heart failure with reduced ejection fraction
HFmrEF: Heart failure with midrange ejection fraction
HFpEF: Heart failure with preserved ejection fraction
